# Intelligent Identification and Prediction of Roof Deterioration Areas Based on Measurements While Drilling

**DOI:** 10.3390/s24237421

**Published:** 2024-11-21

**Authors:** Jing Wu, Zhi-Qiang Zhao, Xiao-He Wang, Yi-Qing Wang, Xiao-Xiang Wei, Zhi-Qiang You

**Affiliations:** School of Energy and Mining Engineering, China University of Mining and Technology (Beijing), Beijing 100083, China; wangxh_1994@163.com (X.-H.W.); wangyiqing0905@163.com (Y.-Q.W.); wxx0621@163.com (X.-X.W.); zqyou0410@163.com (Z.-Q.Y.)

**Keywords:** measurement while drilling, long short-term memory, ABAQUS, mechanical specific energy, roof deterioration areas

## Abstract

During roadway excavation, the presence of roof deterioration zones, such as layered spaces and weak interlayers, significantly affects the stability of the surrounding rock. To achieve timely and effective support for roadways, it is essential to utilize drilling measurement signals obtained during the construction of anchorage holes for the identification and prediction of these deterioration zones. This study systematically investigates the response characteristics of thrust, torque, and Y-direction vibration signals to different combinations of rock layers through theoretical analysis, laboratory experiments, ABAQUS dynamic numerical simulations, and field measurements. The results indicate that these drilling parameters effectively characterize variations in rock structure and strength, with distinct signal features observed particularly in roof deterioration zones. Based on these findings, this paper proposes a deep learning algorithm that employs Long Short-Term Memory (LSTM) recurrent neural networks for classification prediction, along with a random forest algorithm for regression prediction, aimed at the intelligent identification and prediction of roof deterioration zones. The algorithm demonstrates outstanding performance in both laboratory experiments and field tests, achieving a 100% recognition rate for layered spaces and a 96.6% accuracy for identifying deterioration zones, with high accuracy at lower values of mechanical specific energy (MSE). The proposed method provides significant insights for real-time monitoring and control of roof deterioration zones, enhancing the safety and stability of roadway excavations, and serves as a valuable reference for future research and practical applications.

## 1. Introduction

Improving roadway excavation efficiency and reducing roof collapse accidents is crucial for achieving safe, efficient, and intelligent coal mining [1,2]. Research indicates that deterioration in the roof rock layer structure is a primary cause of roof collapses in coal mine roadways supported by bolts [3]. Areas of rock layer deterioration typically include weak interlayers or separation spaces characterized by low hardness, poor cementation, developed fractures, and susceptibility to deformation [4,5]. If deterioration areas are not detected during support operations, anchoring in these areas or partially in overlying stable rock layers can increase the risk of anchor failure and roof collapse. Therefore, accurate and rapid identification of roof deterioration areas in coal roadways is necessary. Current methods for roof detection in coal mines, such as borehole inspection and core sampling [6], suffer from issues such as poor continuity, low real-time capability, high costs, and limited detection range. In contrast, measurement-while-drilling (MWD) offers real-time, precise, and continuous data, making it an effective solution for detecting roof rock layers.

The drilling process involves complex rock fragmentation, during which various feedback signals are generated by the drilling rig and the entire drilling system, depending on the rock strata being drilled or the operating conditions of the rig. The presence of joints, weak planes, or fractured zones in the roof significantly alters the characteristics of the feedback signals from the drilling rig [7,8,9]. Both domestic and international researchers have extensively studied measurement-while-drilling (MWD) and the relationship between drilling signals and rock responses. For example, Fu M. and colleagues [10] observed variations in parameters such as vibration frequency, rotation speed, and torque of the drill rod when drilling through different rock layers. M. Qin [11] found that the vibration and acoustic signal characteristics of rocks correlate well with their mechanical properties. S. S. Peng [12,13] performed laboratory and underground borehole tests using hydraulic anchors with various drilling methods. They used drilling pressure and flow rate to identify rock strength and fractures and developed a geological information system for coal mine roofs. Additionally, parameters such as thrust [14], torque [15], drilling speed [16], and rotation speed [17] respond to rock interfaces, pore fractures, and joints, making them useful for identifying pore fractures and joints [18].

In recent years, deep learning algorithms such as recurrent neural networks (RNN) and convolutional neural networks (CNN) have been increasingly applied. An increasing number of researchers are applying deep learning algorithms to intelligent lithology recognition and prediction [19], handling complex unstructured data [20], developing lithology recognition models [21], and achieving efficient lithology analysis [22,23]. H. Zhang [24] introduced an improved fuzzy clustering algorithm combined with support vector machines (SVMs) to create an intelligent model for lithology recognition. Despite their advantages, CNNs have limitations in extracting features from time series data, which can overlook temporal correlations in drilling parameters. Long Short-Term Memory (LSTM) networks and Gated Recurrent Units (GRUs), as advanced forms of RNNs, overcome these limitations. Q. Sun [25] employed the maximum value of the wavelet variance coefficient for preliminary strata detection and subsequently designed an LSTM-based classification model, which significantly improved the accuracy of lithology recognition.

The identification and prediction of roof deterioration zones during tunnel excavation are crucial for ensuring the stability of the surrounding rock. However, existing methods primarily rely on traditional monitoring techniques, failing to fully leverage real-time data obtained during the construction process. This study aims to explore the dynamic characteristics of roof deterioration zones and their relationship with rock layer combinations through the use of measurement signals obtained while drilling in the anchorage hole construction process. By integrating these real-time measurement signals with the dynamic identification of roof deterioration zones, we provide valuable data support for tunnel support systems. We employ deep learning algorithms based on Long Short-Term Memory (LSTM) networks and Random Forest algorithms to achieve accurate predictions of roof deterioration zones. This approach not only offers a significant theoretical foundation and practical reference for the real-time monitoring and control of roof deterioration but also lays solid groundwork for future research, holding considerable importance for tunnel partition control. Building on existing studies of in-drilling rock layer detection in coal mines, this paper selects three drilling parameters—thrust, torque, and Y-direction vibration signals—as research subjects. We analyze the response characteristics of these parameters to different rock layer combinations to enable effective identification and prediction of roof deterioration zones. To this end, we construct a comprehensive research framework that incorporates theoretical analysis, laboratory experiments, dynamic numerical simulations using ABAQUS, and field measurements.

## 2. Theoretical Study of Drilling Parameters

When drilling anchor holes in the roof, the rock-breaking mechanism of a two-wing PDC (Polycrystalline Diamond Compact) drill bit can be simplified to the combination of rock cutting through the advance movement provided by the drilling rig’s thrust and the rotational motion generated by the torque. This rock fragmentation process can be understood as resulting from the dual types of damage caused by these movements. Additionally, the rock exerts reactive forces and torque on the drill bit, which are fed back to the drilling rig in real time. Consequently, by analyzing the real-time conditions of the drilling rig, one can infer the structure and strength of the rock being cut. Previous studies have demonstrated that thrust, torque, and vibrations in various directions can be monitored during drilling. Given the limitations of experimental equipment and the effectiveness of thrust, torque, and Y-direction vibrations in characterizing the state of the cut rock [26], this paper focuses on these three drilling parameters. To further investigate the qualitative and quantitative impacts of roof deterioration areas on these parameters, a theoretical analysis of their relationship with the cut rock is conducted. To facilitate a better understanding of the symbol parameters discussed in this paper, we have included a symbol notation table listing all the parameters mentioned, as shown in Table 1.

### 2.1. Analysis of Thrust Characteristics

**Figure 1:**
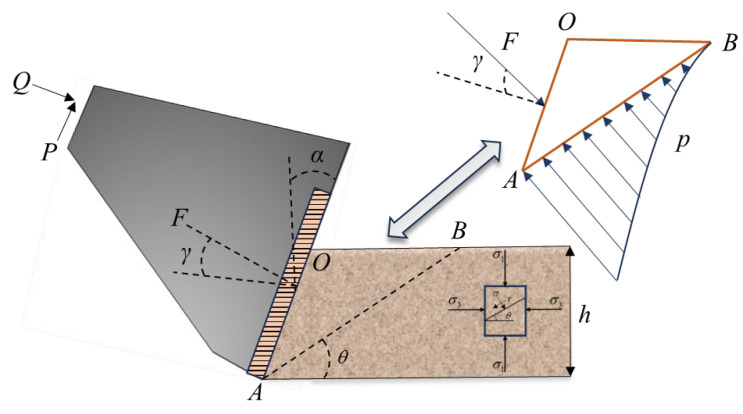
Mechanical model for calculating cutting force of PDC drill bits.

The thrust calculation method, based on the Mohr–Coulomb failure criterion and proposed by Japanese scholar Nishimatsu, is widely employed for analyzing the cutting process of PDC (Polycrystalline Diamond Compact) drill bits on rock formations [27]. This method conceptualizes rock cutting as a planar problem and determines the cutting force from the normal and shear stresses on the shear plane. Figure 1 illustrates the mechanical model used for calculating the cutting force of PDC drill bits.

In the figure, *AB* represents the fracture line created by the drill bit during rock drilling. Within the cutting zone *OAB*, shear cracks initially form and propagate continuously. The point *A* coincides with the tip of the cutting tool. Due to stress concentration, the cutting force *F* is greatest at point *A* and decreases progressively along the line *AB* from *A* to *B*. *α* represents the cutting edge inclination angle; *γ* is the angle between the cutting force *F* and the normal plane of the cutting edge. *Q* and *P* denote the axial thrust provided by the drilling rig and the horizontal cutting force, respectively. 

The cutting block *AOB* is analyzed independently, as shown in Figure 1. Along the straight line *AB*, the force exerted by the rock mass on the cutting block exhibits a power function distribution, with the maximum force occurring at point *A* and gradually decreasing in the direction of *AB*. Representing this power function distributed load with a concentrated resultant force *p*, the load distribution can be expressed as follows:

(1) $$p=p_{0}{\left(\frac{h}{\sin\theta }-x\right)}^{\mu }$$

In this equation, *p*_0_ is the unknown coefficient, a constant; *h* represents the depth of the tool’s penetration into the rock mass; θ is the angle between the straight line *AB* and the cutting direction; *x* is the distance from point *A* to a specific point along the straight line *AB*; and μ is the stress distribution coefficient, which is related to the shape of the cutting edge and remains constant during the cutting process.

Assuming that the direction of the resultant force exerted by the rock mass on the cutting block *AOB* remains constant along the straight line *AB*, we can use integration to determine the resultant force experienced by the cutting body from the rock mass along this line. According to the conditions of static equilibrium, it can be inferred that under limited equilibrium conditions, the external load *F* applied to the cutting body by the tool must be equal to the resultant force exerted by the rock mass on the cutting body along the line *AB*, expressed as follows:

(2) $$F+\int_{0}^{\frac{h}{\sin\theta }}p_{0}(\frac{h}{\sin\theta }-x{)}^{\mu }dx=0$$

By integrating the second term in Equation (2), the unknown coefficient *p*_0_ can be derived as follows:

(3) $$p_{0}=-F(\mu +1){\left(\frac{\sin\theta }{h}\right)}^{\mu +1}$$

By substituting Equation (3) into Equation (1), we can obtain the expression for the unit length load distribution along the straight line *AB* per unit width as follows:

(4) $$p=-F(\mu +1){\left(\frac{\sin\theta }{h}\right)}^{\mu +1}{\left(\frac{h}{\sin\theta }-x\right)}^{\mu }$$

Based on the unit length load distribution along the straight line *AB* per unit width, resultant force *p* can be decomposed into normal and tangential components along the direction of *AB*. This allows for the calculation of shear and normal stresses at point *A*.

(5) $$\left\{\begin{array}{l} \sigma_{A}=-F(\mu +1)\frac{\sin\theta }{h}\sin(\alpha +\gamma +\theta )\\ \tau_{A}=-F(\mu +1)\frac{\sin\theta }{h}\cos(\alpha +\gamma +\theta ) \end{array}\right.$$

Since the failure of the cutting body adheres to the Mohr–Coulomb criterion, the shear and normal stresses at point *A* follow the relationship described by the following equation:

(6) τ=c+σtanφ

By combining Equations (5) and (6) and taking the partial derivative with respect to θ, the cutting force per unit width of the cutting edge can be obtained:

(7) $$F=\frac{2c\cdot v\cdot \cos\varphi }{m\cdot N(\mu +1)\left[\sin(\alpha +\gamma +\varphi )-1\right]}$$

Next, using the geometric relationship between the cutting force *F* and the axial thrust *Q* provided by the drilling rig, the following equation can be derived:

(8) $$Q=F\sin(\alpha +\gamma )\frac{2c\cdot v\cdot \cos\varphi \cdot \sin(\alpha +\gamma )}{m\cdot N(\mu +1)\left[\sin(\alpha +\gamma +\varphi )-1\right]}$$

Assuming the drill bit has a radius *R*, the length of a single row of cutting edges is *L*, and the distance from the unit center to the drill bit center is *r*, and further assuming that the stress per unit width of the drill bit cutting edge is uniform, the total thrust of the drill bit can be obtained by integrating along the direction of the cutting edge:

(9) $$Q_{S}=Q\cdot L\cdot m\frac{2c\cdot v\cdot \cos\varphi \cdot \sin(\alpha +\gamma )\cdot L}{N(\mu +1)\left[\sin(\alpha +\gamma +\varphi )-1\right]}$$

### 2.2. Analysis of Torque Characteristics

The torque experienced by the drill bit not only provides the rotational cutting torque necessary to cut through the rock but also needs to overcome the frictional and resistive torque exerted by the rock on the cutting tool. The frictional and resistive torque can be understood as the component of the rock’s frictional resistance force in the cutting direction, which generates a moment of the drill bit’s center.

According to Equation (8), the horizontal cutting force *P* provided by the drill bit can be similarly derived:

(10) $$P=F\cos(\alpha +\gamma )\frac{2c\cdot v\cdot \cos\varphi \cdot \cos(\alpha +\gamma )}{m\cdot N(\mu +1)\left[\sin(\alpha +\gamma +\varphi )-1\right]}$$

By integrating *P* along the direction of the cutting edge, the cutting moment *M* can be obtained:

(11) $$M=\frac{c\cdot v\cdot \cos\varphi \cdot \cos(\alpha +\gamma )\cdot (2RL-L^{2})}{N(\mu +1)\left[\sin(\alpha +\gamma +\varphi )-1\right]}$$

### 2.3. Analysis of Vibration Characteristics

The basic principle of lateral vibration in a drill rod is as follows: During the drilling process, the resultant force of the reaction from the rock at the bottom of the borehole is transmitted to the drill rod. Under the influence of this reaction force, the drill rod experiences not only a rebound force in the direction opposite to drilling but also recoverable elastic deformation in the lateral direction. This leads to lateral elastic bending of the drill rod, resulting in lateral vibration.

Taking the drilling direction of the drill rod as the *x* axis and the lateral direction as the *y* axis, a force analysis of a differential element of the drill rod during lateral vibration can be performed. According to Newton’s laws, the following equation can be derived [10,28]:

(12) $$\frac{\partial^{4}Y}{\partial x^{4}}+\frac{F\sin\omega (t)}{EI}\frac{\partial^{2}Y}{\partial x^{2}}-\Omega^{2}\frac{\rho A}{EI}Y=0$$

Here, *Y* represents the lateral vibration radius of an infinitesimal segment *dx* of the drill rod, ω(t) denotes the amplitude of the response force as a function of time, and *EI* corresponds to the bending stiffness of the drill rod. Additionally, ρ refers to the average mass per unit length of the drill rod, *A* is the cross-sectional area of the drill rod, and Ω signifies the natural frequency of the drill rod. Since *EI*, *f*, ρ, Ω, and *A* are all constants for a particular drill pipe, the transverse vibration radius *Y* of the drill pipe can be abbreviated in the form of *Y* = *Y*(*x*, *t*, *F*), and the transverse vibration velocity of the drill pipe can be obtained by taking the 1st derivation of *Y*(*x*, *t*, *F*) with respect to *t*:

(13) $$\nu_{Y}=\frac{\partial Y(x,t,F)}{\partial t}$$

In summary, thrust and torque are associated with the strength parameters of the drilled material, such as cohesion and internal friction angle. The amplitude of lateral vibration is related to the cutting force, which is also connected to these strength parameters. Roof deterioration zones typically exhibit weaker strength parameters compared to more stable rock layers. Consequently, to better understand the relationship between these drilling parameters and the strength indicators of the drilled material, as well as the response patterns of these parameters in deterioration zones, further research and verification are required.

## 3. Response Patterns of Drilling Signals in Degraded Zones

Accurately identifying and predicting degraded zones in the roof during tunnel construction in mines is crucial for ensuring construction safety. This chapter presents a comprehensive study of drilling signal response patterns in degraded zones through experiments and numerical simulations. The analysis focuses on three primary drilling parameters: thrust, torque, and Y-direction vibration signals. It systematically examines their response characteristics across various rock layer combinations and investigates the correlation between signal variations and rock layer degradation.

**Figure 2:**
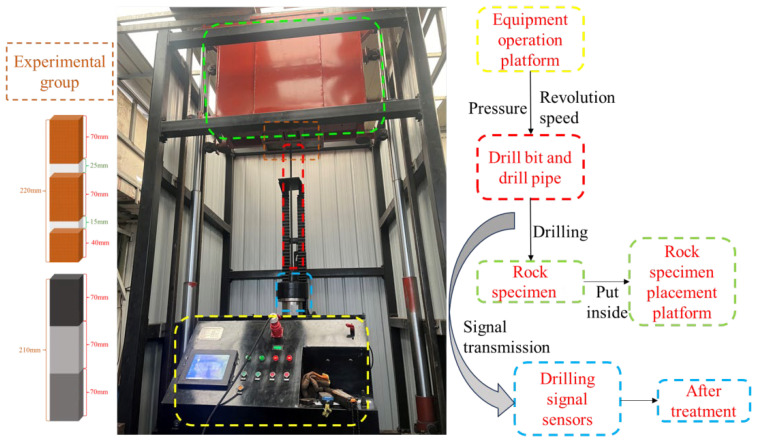
Drilling experiment equipment diagram.

As illustrated in Figure 2, our research team developed a drilling detection system that utilizes a control platform to regulate pipeline pressure for the ascent and rotation of the drill rod. The system enables the drill bit to cut through rock specimens using both advancing and rotational movements. To simulate real-world conditions, two types of rock layer combination experiments were designed. The first type involves combinations of red sandstone with separation spaces of varying widths, specifically 15 mm and 25 mm gaps between each specimen. The second type consists of concrete specimens of different strengths, C30, C20, and C10, where the numbers represent the uniaxial compressive strength of the concrete.

**Figure 3:**
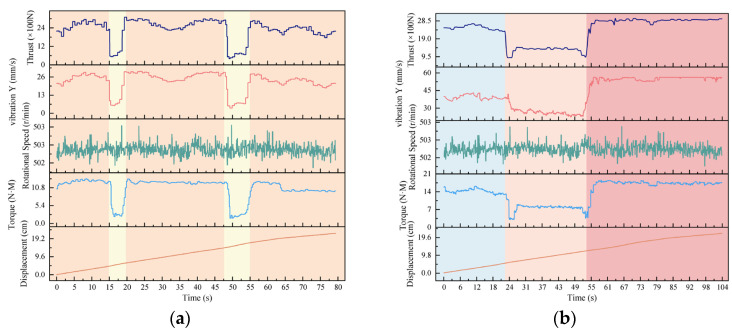
Drilling signal diagram. (**a**) Combinations of rock layer and separation; (**b**) combinations of concrete.

During drilling, data on torque, thrust, rotational speed, and Y-direction vibration of the drill rod were collected and recorded using drilling signal sensors. These sensors operated at a frequency of 60 Hz, sampling every 0.02 s. To mitigate signal interference, the collected data were preprocessed by trimming signals recorded before the drill bit reached the bottom interface of the specimens and during retraction. Due to the large volume of data, unclear frequency structure, and significant interference, wavelet denoising was employed to reduce noise and refine the signals. The processed signal accumulation diagram is shown in Figure 3. The different colored boxes in the actual picture of the equipment represent the different components of the different drilling systems and are consistent with the different colored boxes on both sides.

The drilling equipment maintained a nearly constant rotational speed with only minor fluctuations, so a detailed analysis of rotational speed is not included in this study. Figure 3a presents the analysis of rock layer combinations with interlayer spaces, revealing several key observations. From 0 to 15 s, the drill bit targeted a 40 mm red sandstone layer. During this period, the thrust and torque signals remained relatively stable due to minimal constraints on the drill rod, while the Y-direction vibration signal exhibited some fluctuations. From 15 to 20 s, when drilling a 15 mm interlayer, the thrust, torque, and Y-direction vibration signals all experienced significant drops before stabilizing. At the interface between the interlayer and the subsequent red sandstone layer, these signals increased, forming an “inverted trapezoid” distribution. From 20 to 47 s, the target was a 70 mm red sandstone layer, where the thrust, torque, and Y-direction vibration signals maintained similar amplitudes and values to those recorded during drilling of the initial red sandstone layer. From 47 to 55 s, the target was a 25 mm interlayer, where the signals again followed a three-phase process of decline, stabilization, and subsequent rise, resembling the previous interlayer’s “inverted trapezoid” distribution. Finally, from 55 to 80 s, the drill bit targeted another 70 mm red sandstone layer (with 5 mm remaining undrilled). Although there was a slight weakening in the signal toward the end, the overall trend and amplitude remained consistent with the red sandstone drilling pattern. Figure 3b further illustrates the signal analysis results for different strength rock layer combinations. From 0 to 22 s, the target was a 70 mm C20 concrete specimen, during which the Y-direction vibration signal displayed significant fluctuations while the thrust and torque trends remained consistent. From 22 to 51 s, due to the placement of the concrete specimens, a gap existed between the C20 and the central C10 concrete specimens, causing substantial drops in torque and thrust over a short interval. As the drill bit entered the C10 concrete specimen, the signals increased and stabilized, though at lower levels compared to the C20 concrete drilling signals. From 51 to 117 s, the target was a C30 concrete specimen, where thrust, torque, and Y-direction vibration signals showed significant increases, reaching their maximum values during the drilling process. The combined drilling signals displayed a “medium-weak-strong” pattern, consistent with the uniaxial compressive strengths of the different concrete specimens. Overall, this signal analysis indicates that thrust, torque, and Y-direction vibration effectively characterize variations in rock strength and deterioration zones.

**Figure 4:**
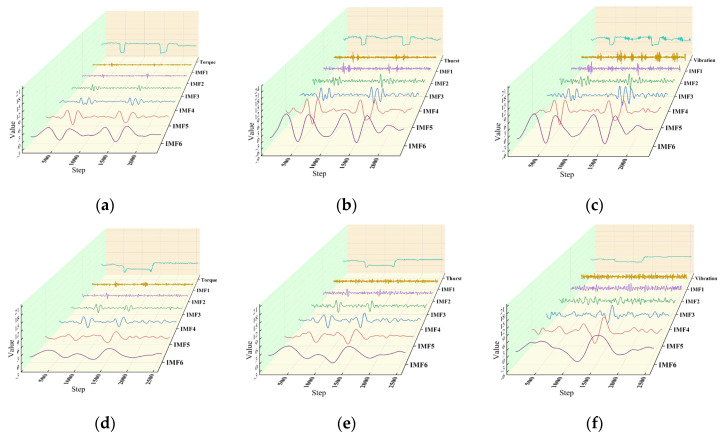
Drilling signal complementary ensemble empirical mode decomposition diagram. (**a**) Separation layer (torque); (**b**) separation layer (thrust); (**c**) separation layer (vibration); (**d**) rock strata (torque); (**e**) rock strata (thrust); (**f**) rock strata (vibration).

Waveforms are composed of multiple harmonics, so analyzing the frequency characteristics of drilling signals requires decomposing the raw data into intrinsic mode functions (IMFs) across different time scales. In 1998, Norden E. Huang [29] introduced the Empirical Mode Decomposition (EMD) method, which breaks down complex signals into a sum of mode functions with progressively higher frequencies. By applying the Hilbert transform to these mode functions, the signal’s frequency spectrum and energy distribution can be obtained. However, EMD has limitations, such as mode mixing and endpoint effects. To overcome these issues, Yeh [30] proposed the Complementary Ensemble Empirical Mode Decomposition (CEEMD) method, which enhances EMD by adding pairs of white noise with opposite polarities to the signal. This approach mitigates mode mixing and reduces errors caused by white noise. In this study, CEEMD is employed to analyze thrust, torque, and Y-direction vibration signals for rock layers with interlayer spaces and varying strengths. As shown in Figure 4, CEEMD’s intrinsic mode functions exhibit high-frequency oscillations at interlayer positions, whereas the functions remain relatively stable during red sandstone drilling. Among different strength rock layer combinations, IMF5 and IMF6 show larger errors, while other components effectively represent variations in rock strength. Therefore, the characteristics of CEEMD intrinsic mode functions can be used as supplementary indicators for identifying deterioration zones.

**Figure 5:**
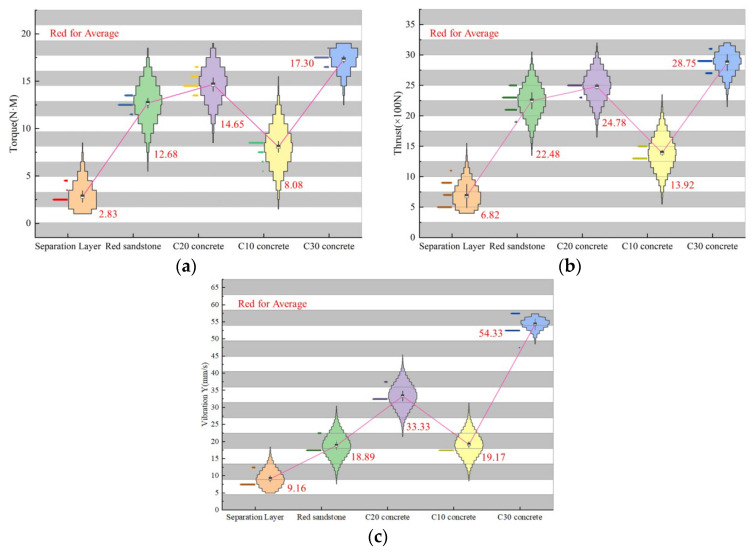
Drilling signal violin diagram. (**a**) Torque signal; (**b**) thrust signal; (**c**) vibration signal.

To better analyze the differences between various drilling targets, we extracted thrust, torque, and Y-direction vibration signals from two datasets and created violin plots, as shown in Figure 5a. The torque values, arranged from smallest to largest, are as follows: interlayer space, C10 concrete specimen, red sandstone specimen, C20 concrete specimen, and C30 concrete specimen. The data distribution densities are relatively concentrated: torque in interlayer spaces ranges from 2.1 to 3.3 N·m, in red sandstone from 11.7 to 13.5 N·m, in C10 concrete from 5.8 to 8.4 N·m, in C20 concrete from 13.4 to 16.1 N·m, and in C30 concrete from 16.5 to 17.9 N·m. The significant variation in torque among different drilling targets makes it a key indicator for identifying deterioration zones. Similarly, as illustrated in Figure 5b, thrust values are arranged in the same order: interlayer space, C10 concrete specimen, red sandstone specimen, C20 concrete specimen, and C30 concrete specimen. Thrust in interlayer spaces ranges from 439 to 910 N, in red sandstone from 1940 to 2270 N, in C10 concrete from 1260 to 1340 N, in C20 concrete from 2390 to 2520 N, and in C30 concrete from 2670 to 2925 N. The considerable variation in thrust among drilling targets further confirms its importance as an indicator for deterioration zones. Figure 5c shows the Y-direction vibration values, also arranged from smallest to largest: interlayer space, C10 concrete specimen, red sandstone specimen, C20 concrete specimen, and C30 concrete specimen. Y-direction vibration in interlayer spaces ranges from 8.9 to 11.1 mm/s, in red sandstone from 15.9 to 20.2 mm/s, in C10 concrete from 17.8 to 20.6 mm/s, in C20 concrete from 31.2 to 35.8 mm/s, and in C30 concrete from 48.9 to 55.4 mm/s. The substantial differences in Y-direction vibration across drilling targets highlight its role as a critical indicator for identifying deterioration zones.

**Figure 6:**
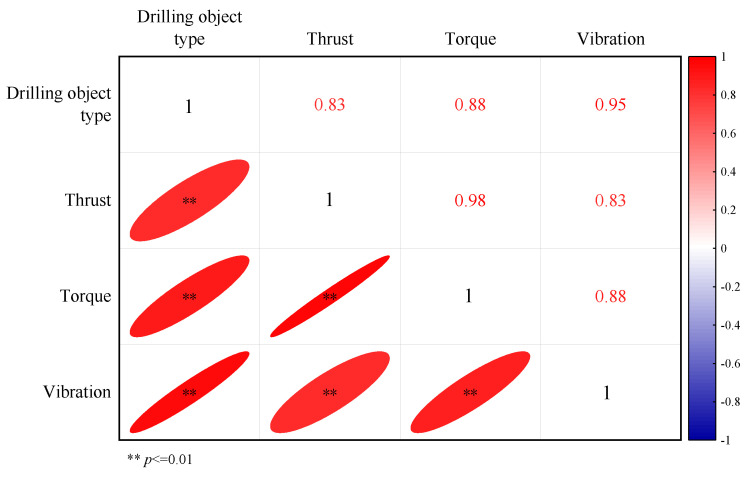
Correlation index analysis diagram.

To analyze the sensitivity indicators between the types of drilling objects and the three drilling parameters, the drilling objects were categorized numerically, establishing a correspondence between the drilling objects and the drilling parameters. A correlation analysis was conducted on the generated data, with the results presented in Figure 6. The correlation coefficient between thrust and the type of drilling object was found to be the highest at 0.88, indicating that thrust is a strong indicator of changes in the drilling object. This was followed by torque and vibration. Among the drilling parameters, the correlation between torque and thrust was the strongest, suggesting that thrust and rotational cutting force exhibit a coordinated and synchronous variation. In summary, there is a positive correlation between the type of drilling object and the drilling parameters, as well as a positive correlation among the three drilling parameters themselves.

**Figure 7:**
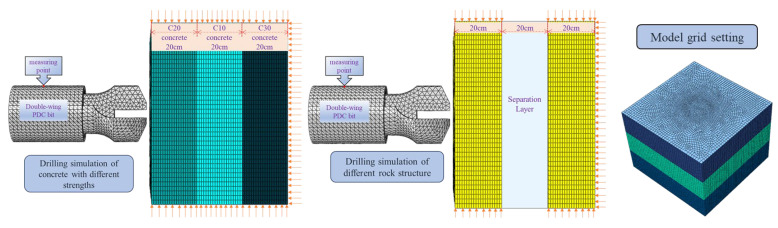
Numerical simulation model diagram.

To better validate the response pattern of drilling signals to the layered space, a model of a mining two-wing PDC drill bit was established using SolidWorks software, and two design schemes were created, as shown in Figure 7. These schemes consider combinations of rock layers and layers of varying strengths. The right side of Figure 7 illustrates different structural combinations of rock layers, with a 20 cm thick gap left between the two rock mass models. The dimensions of the red sandstone specimens are 50 cm × 20 cm × 50 cm. The middle view of Figure 7 displays the combination of rock layers with different strengths, specifically C20 concrete, C10 concrete, and C30 concrete, with the same specimen dimensions of 50 cm × 20 cm × 50 cm, while the drill bit moves in the positive Y direction. After the model was constructed, it was imported into the ABAQUS numerical simulation software for computation. During the drilling process, the drill bit is assumed to be rigid and not subject to wear due to drilling; thus, it is simplified as a rigid body, and the surrounding rock mass is completely fixed. Since the rock-breaking process primarily occurs in the area of the rock model corresponding to the direction of the PDC drill bit, we performed mesh refinement in a specific region of the rock specimen aligned with the drilling direction to better simulate and calculate the PDC drilling and rock-breaking process. In contrast, locations further away from the PDC drill bit’s drilling direction were not subjected to mesh refinement. In this experiment, the refined mesh region predominantly utilized hexahedral elements with a mesh size of 1 mm. In the drill feed direction (Y direction), the model was further refined with a mesh edge length of 2 mm; the unrefined mesh at the far end also primarily employed hexahedral elements. The right side of Figure 7 provides a schematic representation of the model’s mesh partitioning. To effectively simulate the deformation and failure of the rock mass, a linear elastic model was employed for the elastic phase of the rock mass, while the Drucker–Prager model was utilized for the plastic phase. The parameters for the rock mass are detailed in Table 2. The distance between the drill bit and the rock specimen was maintained at 2 mm, with a reference point set at the drill bit’s end, to which a velocity of 60 cm/s and a rotational speed of 51 rad/s were applied. The analysis time step was set to 1 s. Since all data on the rigid body are equal, any node at the top of the drill bit was selected as a measurement point to extract the torque and thrust parameters at each frame, thereby validating the response pattern of the drilling signal.

**Figure 8:**
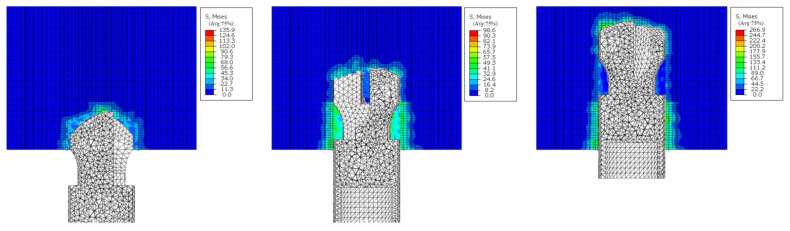
Mises stress distribution of different rock strata combinations in drilling process.

**Figure 9:**
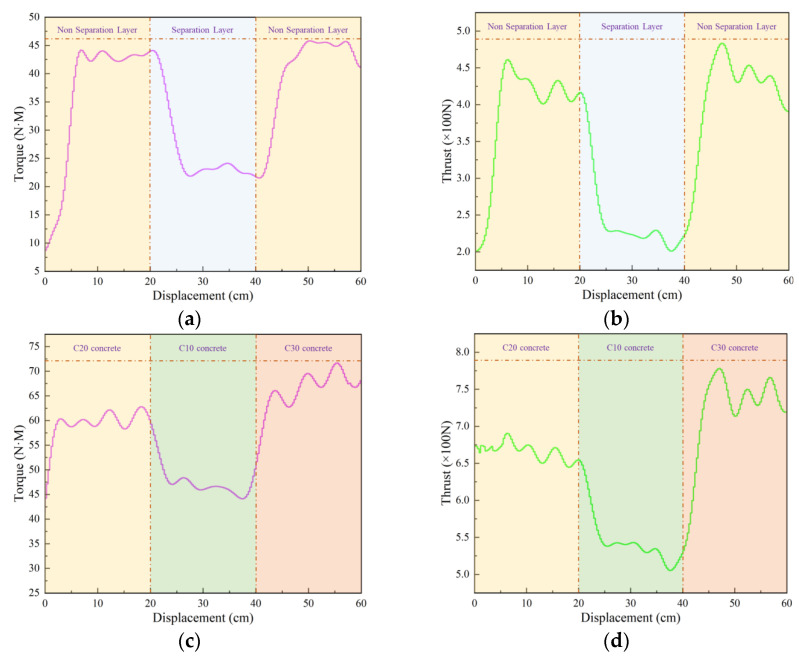
Drilling signal curve diagram. (**a**) Separation layer (torque); (**b**) separation layer (thrust); (**c**) tock strata (torque); (**d**) rock strata (thrust).

Figure 8 is the Mises stress distribution under different strength rock strata combinations obtained by simulation. From the diagram, it can be seen that the stress distribution pattern is consistent with the strength of the drilled object. With the increase in the strength of the drilled rock, the Mises stress also increases. In order to further analyze the response relationship between rock structure, rock strength, and drilling parameters, Figure 9 illustrates the drilling signals at different interfaces, with distinct colors demarcating the boundaries between various drilling targets. Figure 9a,b present the thrust and torque signals for the combination of red sandstone and interlayer space. At these interfaces, a pronounced change in the drilling signals is observed: both thrust and torque experience a sharp decline when transitioning from red sandstone to the interlayer space and a sudden increase when transitioning back from the interlayer space to red sandstone. Figure 9c,d display the thrust and torque signals for concrete specimens of varying strengths. Similar to the interlayer space scenario, abrupt changes are evident at the boundaries between different concrete strengths. The drilling signals exhibit a “medium-weak-strong” pattern, with slight amplitude variations noted in drilling targets with higher strength.

**Figure 10:**
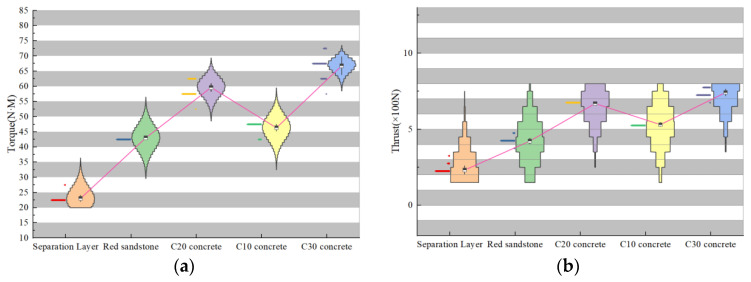
Simulated drilling signal violin diagram. (**a**) Torque signal; (**b**) thrust signal.

To better characterize the variation in drilling parameters across different drilling targets, we extracted thrust and torque data from two scenarios and combined them to create violin plots, as illustrated in Figure 10. To more accurately represent the effect of rock strength changes on drilling parameters, the strength of the C10 concrete specimen was set slightly higher than that of the red sandstone specimen. This setup ensures that aside from the differences in drilling parameters between C10 concrete and red sandstone, the trends observed in other scenarios are consistent with laboratory experiments. The thrust and torque values, ordered from smallest to largest, are as follows: interlayer space, red sandstone, C10 concrete, C20 concrete, and C30 concrete. This analysis confirms that drilling signals effectively identify changes in rock strength and detect deterioration zones based on rock strength.

## 4. Intelligent Identification and Prediction of Deterioration Zones

**Figure 11:**
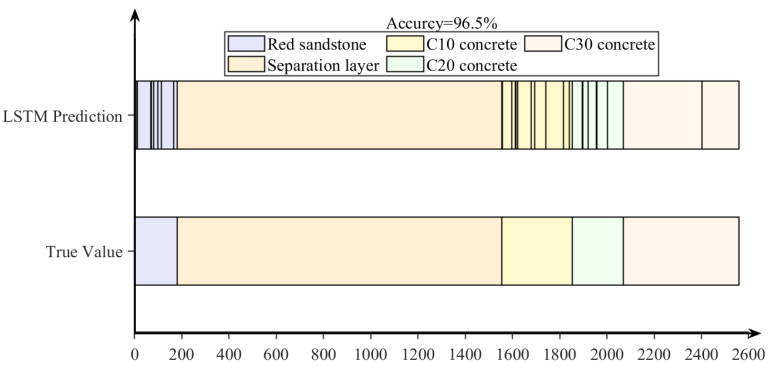
LSTM prediction results diagram.

**Figure 12:**
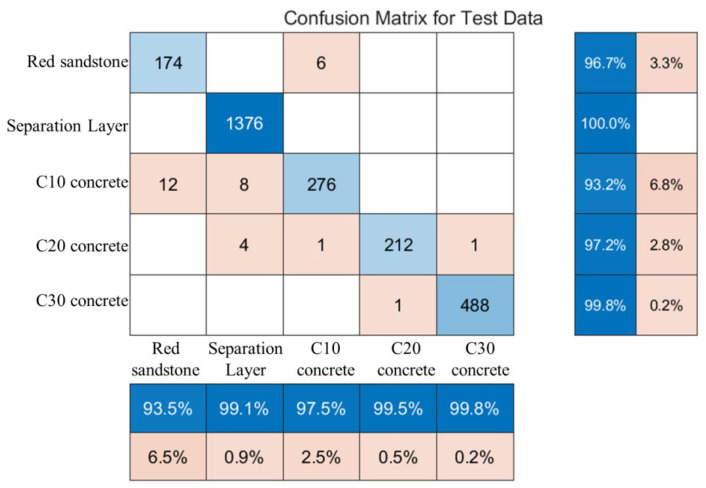
Confusion matrix diagram.

Based on the previous analyses, thrust, torque, and Y-direction vibration are highly sensitive to changes in drilling targets and rock strength. In mining operations, roof deterioration zones primarily consist of interlayer spaces and weak rock layers. Timely and accurate control of the development and damage of these deterioration zones is crucial, making real-time identification and intelligent prediction essential. Deep learning algorithms are currently prevalent in data processing, recognition, and prediction tasks. Among these, the Long Short-Term Memory (LSTM) network, which incorporates a gating mechanism, effectively mitigates the vanishing gradient problem, allowing it to process longer sequence data. Compared to traditional Recurrent Neural Networks (RNNs), LSTMs exhibit superior memory performance, enabling the retention of distant contextual information when handling sequential data [31]. Additionally, LSTMs are sensitive to time, allowing them to learn patterns and features within temporal data. This capability provides LSTMs with advantages in tasks such as time series forecasting and signal processing. However, the computational complexity of LSTMs can be substantial and time-consuming, and their intricate internal mechanisms can make the decision-making process of the network less intuitive and harder to interpret. Given that drilling signals possess temporal characteristics and a large volume of data samples is generated during the experimental process, this study proposes a recurrent neural network based on the LSTM approach to achieve intelligent identification and prediction of roof deterioration zones according to drilling parameters. In the initial experimental design, four sets of experiments were developed: two sets involving layered spaces and two sets comprising different combinations of concrete specimens with varying strengths. One group was utilized to extract signal features as the training dataset, while the other group served as the testing dataset to validate the accuracy of the LSTM-based intelligent identification method for drilling parameters related to the drilled objects. Figure 11 and Figure 12 present the comparison of prediction accuracy for the testing dataset and the confusion matrix, respectively.

Figure 11 illustrates the classification predictions of drilling targets using the LSTM recurrent neural network, with a prediction accuracy of 96.5%. The vertical lines in the LSTM predictions represent regions predicted as different drilling targets. Due to the large number of samples, these lines are densely packed. The density of these lines indicates that the LSTM method performs effectively in identifying interlayer spaces and C30 concrete specimens, though it exhibits slightly reduced accuracy for medium-strength rock specimens. Figure 12 shows that the recognition rate for interlayer spaces is 100%, while the lowest recognition rate for other drilling targets is 93.2%. Based on the strengths of the drilling targets, this study defines red sandstone specimens, interlayer spaces, and C10 concrete specimens as deterioration zones. The method achieved a 96.6% accuracy rate in identifying these deterioration zones. Overall, the proposed LSTM-based intelligent recognition method demonstrates strong performance in identifying and predicting drilling targets using laboratory data. However, given that anchoring holes are constructed in underground tunnels with more complex geological and stress conditions, further validation is required to apply this method effectively in real field conditions for controlling roof deterioration zones.

**Figure 13:**
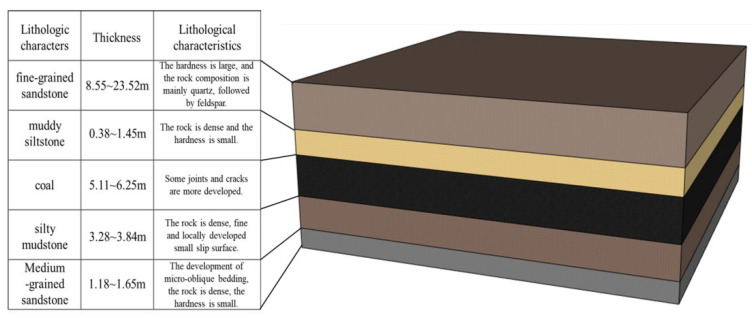
Rock stratum histogram.

**Figure 14:**
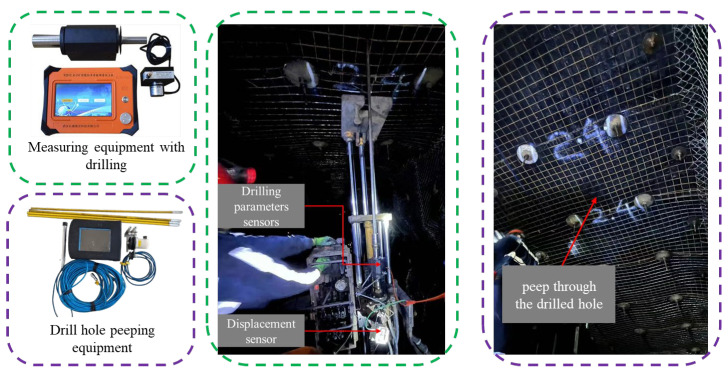
Field test diagram.

To further validate the intelligent recognition and prediction based on drilling parameters, this study conducted real-time drilling detection at the Dahaize coal mine’s transportation tunnel in Shaanxi. The current rock column diagram of the coal seam is depicted in Figure 13. The tunnel is excavated along the floor, with approximately 1 m of coal seam remaining at the roof. Above the coal seam lies a layer of muddy siltstone with low strength, and above that is a layer of fine-grained sandstone, which forms the main roof with higher strength. Within a 3 m range, the roof will exhibit at least three types of rock lithology changes, facilitating a clearer representation of changes in drilling signals relative to different drilling targets. During the construction of the roof anchoring holes, drilling parameter sensors were installed between the drill rig and the drill pipe to capture real-time values of thrust, torque, and Y-direction vibration. Displacement sensors were mounted on the rig’s base to measure changes in drilling displacement over time. The field experiment setup is shown in Figure 14.

**Figure 15:**
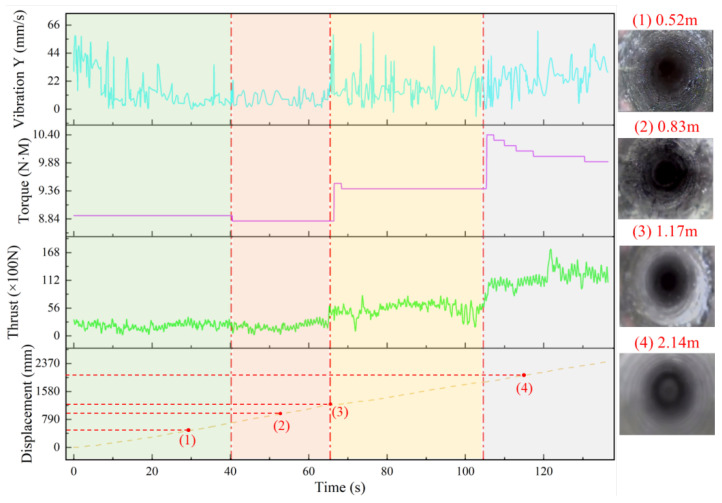
Field drilling signal diagram.

After completing the real-time drilling detection, borehole inspections were conducted to correlate the drilling signals with actual changes in the drilling targets. Figure 15 illustrates the findings from these inspections:

Location (1): The drilling target is coal with smooth and intact borehole walls. The drilling signal curve indicates that both thrust and torque remain relatively constant and low. Due to the high power of the drill and the initial drilling stage, the vibration amplitude is high but stabilizes and fluctuates minimally later on.

Location (2): The borehole inspection reveals that the drilling target is still coal, but the borehole walls exhibit some fractures, and the coal is more fragmented. The corresponding drilling signal shows a slight decrease in thrust and torque, with a small decrease in vibration amplitude. However, these changes are not prominent due to fluctuations within a certain range.

Location (3): The inspection clearly identifies the coal–rock interface, with the drilling target being muddy siltstone. The drilling signal curve reflects a significant increase in thrust and torque, along with a noticeable rise in vibration amplitude.

Location (4): The rock appears gray with smooth, intact borehole walls, and the drilling target is fine-grained sandstone. The drilling signal curve shows significant increases in both torque and thrust, with a smaller change in vibration amplitude, although the amplitude increases further. Combining the drilling signals with borehole inspection results and the rock column diagram, it is evident that thrust, torque, and Y-direction vibration parameters effectively describe changes in the underground roof layers. These parameters can clearly identify deterioration zones in the roof. Additionally, the drilling parameters validate the effectiveness of the LSTM neural network in predicting rock strength trends and deterioration zones.

**Figure 16:**
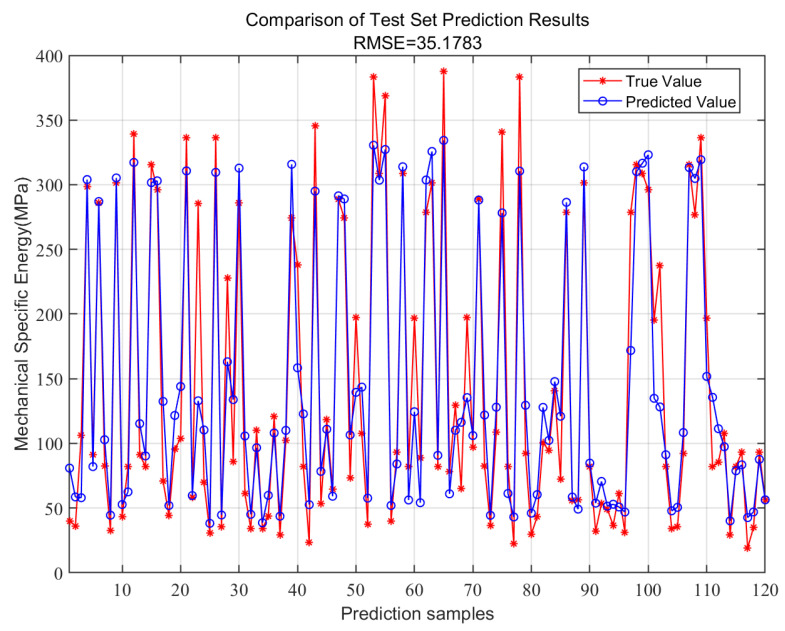
The comparison diagram between true value and predicted value of MSE.

The previous LSTM model, which was used for classification prediction, proved inadequate for precise on-site engineering applications due to its lack of accuracy in intelligent prediction. To address this issue, it is essential to identify a value that represents the trend of changes in the drilling target’s characteristics. Due to the correlation between the mechanical specific energy of the drill bit and the uniaxial compressive strength of the rock mass, the mechanical specific energy can, to some extent, serve as a proxy for rock strength [32]. Thus, predictions of mechanical-specific energy during the drilling process can replace direct strength assessments of the drilled material. In this study, 300 data points were extracted to calculate the mechanical-specific energy of the drill bit during the ongoing drilling process. Out of these, 180 data points were designated as the training set, while 120 data points were used for testing. The thrust, torque, and Y-direction vibration were utilized as input features for regression predictions of the mechanical specific energy for each data point. Given that the LSTM model is less effective for predictions with limited data and exhibits lower accuracy for non-sequential data samples [33], this paper employs the random forest algorithm to predict mechanical specific energy during drilling. The predicted results are illustrated in Figure 16. Due to the complex field testing environment and the interference of various factors, the root mean square error (RMSE) of the mechanical-specific energy predictions was found to be 35.18, indicating some level of error in the numerical predictions. However, the algorithm demonstrated good performance in capturing the trends of mechanical-specific energy changes, particularly at lower values where identification accuracy was notably high. Therefore, it can be concluded that the random forest algorithm effectively utilizes drilling parameters to identify and predict lower-strength coal rock masses, thereby aiding in the recognition of roof deterioration areas.

## 5. Conclusions

This study establishes a theoretical model that correlates various drilling parameters with rock structure and strength. By combining numerical simulations and laboratory experiments, we integrate drilling measurement signals with the dynamic identification of roof deterioration zones, providing real-time data support for roadway support systems. Furthermore, we employ deep learning and machine learning algorithms for the intelligent identification and prediction of roof deterioration zones, leading to the following conclusions:

(1) All drilling parameters exhibit a positive correlation with rock strength. Among these parameters, thrust is the most indicative of changes in rock structure and strength, with a correlation coefficient of 0.88. Torque and Y-direction vibration also show positive correlations, with similar trends observed across different drilling targets. The sequence of values from smallest to largest is discontinuity < red sandstone < C10 concrete < C20 concrete < C30 concrete.

(2) This study classifies discontinuities, red sandstone, and C10 concrete as deterioration zones. Laboratory experiments and ABAQUS dynamic simulations reveal significant decreases in thrust, torque, and Y-direction vibration at the boundaries of these zones. On-site tests in fractured coal and weak roof sections further confirm these findings, showing similar reductions in these parameters at boundaries. These results indicate that all drilling parameters are effective in responding to deterioration zones in the roof.

(3) The LSTM-based classification prediction for roof rock layer identification achieves an accuracy of 96.5%, with a recognition rate of 100% for layered spaces and an accuracy of 96.6% for identifying deterioration zones. Additionally, the regression prediction of mechanical-specific energy based on the random forest algorithm exhibits some discrepancies in numerical predictions; however, it effectively captures the trend of changes in specific energy. Notably, at lower specific energy values, the recognition accuracy is particularly high, allowing for effective identification of roof deterioration zones.

Based on this, this study has identified the response patterns of drilling parameters in roof deterioration zones and proposed a method for the identification and prediction of these zones based on drilling parameters. However, due to the limitations of experimental conditions and the author’s expertise, the following aspects require improvement in future research:

1. Additional drilling parameters, such as drilling speed and vibrations in the X and Z directions, can also characterize variations in rock structure and strength. Establishing dynamic relationships between these parameters and roof deterioration zones is necessary to further enhance identification accuracy.

2. In the underground environment, various stress forms, including self-weight stress, tectonic stress, and mining-induced stress, overlap during the excavation process. The patterns and numerical values of drilling parameters may vary under different stress conditions, and the morphology of roof deterioration zones will change accordingly. Future research on the response patterns of drilling parameters in roof deterioration zones under varying stress states is also of significant importance.

3. Currently, the dataset is insufficiently large, and there are certain deficiencies in the selection of corresponding algorithms. Future studies should expand the sample size of experimental and field data to compare the identification accuracy of different algorithms based on drilling parameters for roof deterioration zones in order to select the most suitable algorithm.

## Figures and Tables

**Table 1:** Symbol notation table.

Symbol	Meaning	Symbol	Meaning
*Q*	Axial thrust provided by the drilling rig	*m*	Number of cutting edge rows on the drill bit
*P*	Horizontal cutting force provided by the drilling rig	*R*	Radius of the drill bit
*F*	Cutting force	*L*	Length of a single row of cutting edges
*α*	Rake angle of the cutting edge	*v*	Cutting speed
*γ*	Angle between the cutting force F and the normal plane of the cutting edge	$\sigma_{A}$	Normal stress at point A
θ	Angle between line AB and the cutting direction	$\tau_{A}$	Shear stress at point A
μ	Stress distribution coefficient on the cutting surface	*Q_S_*	Resultant thrust of the drill bit
*h*	Depth of cut into the rock	*r*	Distance from the center of the unit to the center point of the drill bit
φ	Internal friction angle of the rock	ω(t)	Amplitude of the response force, varying over time
*c*	Cohesion of the rock	*EI*	Bending stiffness of the drill rod
*N*	Rotational speed of the drill bit	ρ	Average mass per unit length of the drill rod
*f*	Natural frequency of the drill rod	*A*	Cross-sectional area of the drill rod
*M*	Cutting torque	*Y(X)*	Lateral vibration radius of the drill rod
*p*	Force exerted by the rock mass on the cutting body	*AB*	Fracture line generated during the drilling process
*P_0_*	Undetermined coefficient	Ω	Natural frequency of the drill rod

**Table 2:** Rock parameters.

Lithologic Characters	Elastic Modulus/GPa	Dilation Angle/(°)	Density/(g/cm^3^)	Poisson	Flow Stress Ratio	Compression Yield Stress/MPa	Friction/(°)
Red sandstone	3.9	46.1	2400	0.25	0.778	13	28.4
C10 concrete	5.3	48.5	2420	0.22	0.778	17	29.7
C20 concrete	7.1	51.2	2600	0.18	0.778	25	32.2
C30 concrete	9.5	53.6	2710	0.13	0.778	34	34.7

## Data Availability

The original contributions presented in this study are included in the article. Further inquiries can be directed to the corresponding authors.
